# Queries Respecting Albinos

**Published:** 1833-02

**Authors:** E. G. Ballard

**Affiliations:** Islington.


### ALBINOS.

### *Queries respecting Albinos.* By E. G. Ballard, Esq. Islington.

There is, perhaps, no phenomenon more singular in physiology than that of Albinos; the following account of which, and the queries suggested by it, may possibly lead to some investigation on this curious and interesting subject.

In Saussure's "Voyages dans les Alpes," is an account of two boys at Chamouni, who were called Albinos. The skin was perfectly white, like milk, as well as their hair. In the eyes, both the iris and pupil were red. This he accounts for by stating, that probably the interior membranes of the eye were destitute of the uvea and of that black mucus which should line them.

The Albinos of Africa differ in nothing from these, but in their having the features of blacks.

M. Blumenbach agrees with the opinion of the cause of the redness of the eyes, and says that the different colours of the eyes, as well as the strength of them, as people with light eyes cannot bear a strong light, suits very well with northern nations during their long twilight; while, on the contrary, the darkness of the eyes of blacks enables them to support the splendor of the sun's beams in the torrid zone. As to the connexion of the colour of the eyes and the whiteness of the skin and hair, he refers it to similarity of structure. He asserts, that this black mucus is formed only in the delicate cellular texture, which has numerous blood-vessels contiguous to it, but contains no fat, like the inside of the eye, the skin of negroes, the spotted palate of domestic animals, &c.; and, lastly, he says, the colour of the hair generally corresponds with that of the iris.

M. Buzzi, surgeon to the hospital at Milan, in a very interesting memoir, demonstrates, by dissection, what Blumenbach had only conjectured. A peasant, of about thirty years of age, died at the hospital of Milan: his body, being exposed to view, was exceedingly remarkable by the uncommon whiteness of the skin, hair, beard, &c. M. B., who had long wished to dissect such a subject, immediately seized upon this: he found the iris of the eyes completely white, and the pupil of a rose colour. On dissecting them, with the greatest possible care, they were found entirely destitute of the black membrane which anatomists call the uvea. It was not to be found either behind the iris or under the retina. Within the eye there was only found the choroid coat, extremely thin, and tinged of a pale red colour, by vessels filled with discoloured blood. What was most extraordinary, the skin, when detached from different parts of the body, seemed also divested of the rete mucosum: maceration did not discover the least vestige of it, not even in the wrinkles of the abdomen, where it is most abundant and most visible.

M. B. likewise accounts for the whiteness of the skin and hair, by the absence of the rete mucosum, which, he says, gives the colour to the cuticle, and to the hairs which are scattered over it. In support of this, he adduces a well-known fact, that, if the skin of the blackest horse be by chance destroyed in any part of the body, the hair that afterwards grows there is always white, as is the skin, because the rete mucosum, which gave both their colour, is never regenerated in the skin.

These arguments, which are extracted from the "Encyclopædia Londinensis," seem conclusive, and are necessary to the introduction of the following queries, which suggest themselves on the subject.

1. As the rete mucosum is evidently as much a secretion from the mass of blood as any of the other secretions, is it not, like many of them, lessened in quantity by disease? and is not this one cause of the paleness attendant on many acute and constitutional diseases, as inflammation of the lungs, consumption, &c.?

2. May not the same deficiency of this secretion occasion the hair in very old people to turn gray;* considering that the constant renewal of the human frame is going on, and that the hair daily, though perhaps imperceptibly decays, or is reduced by cutting; and that in itself being tubular, may, through the orifice, when first made, discharge, in some degree, the colouring matter it has imbibed through the pores of its roots, which no longer affording a supply of the rete mucosum, the oxygen in the atmosphere may operate upon the exterior membrane which constitutes the hair, and reduce it to whiteness?

But this involves another question, viz. whether the oxygen in the atmosphere operates upon the colouring matter of the human frame, as it does on that of vegetables?

3. How is it, if, as the above statement suggests, the black membrane in the eye, (or, as it is called, the uvea,) be of the same nature as the rete mucosum, that a white dog, whose skin under the hair is of the same colour nearly as that of the human body, and, by the whiteness of the hair and fairness of the skin, may be supposed to want the rete mucosum, that the eyes of such a dog are not red but hazle, when a white rabbit, under the same circumstances, shall have red eyes?

* On examining a gray hair from my own head, I found the root devoid of colouring matter, and the tube perfectly white, transparent, and slightly corrugated, as if dead.

